# Environmental Factors Associated With Type 1 Diabetes

**DOI:** 10.3389/fendo.2019.00592

**Published:** 2019-08-28

**Authors:** Susanna Esposito, Giada Toni, Giorgia Tascini, Elisa Santi, Maria Giulia Berioli, Nicola Principi

**Affiliations:** ^1^Pediatric Clinic, Department of Surgical and Biomedical Sciences, Università degli Studi di Perugia, Perugia, Italy; ^2^Università degli Studi di Milano, Milan, Italy

**Keywords:** diet, gut microbiota, immunotherapy, infections, primary prevention, type 1 diabetes

## Abstract

Type 1 diabetes (T1D) is a chronic autoimmune disorder that leads to progressive pancreatic ß-cell destruction and culminates in absolute insulin deficiency and stable hyperglycaemia. It is very likely that environmental factors play a role in triggering islet autoimmunity. Knowing whether they have true relevance in favoring T1D development is essential for the effective prevention of the disease. Moreover, prevention could be obtained directly interfering with the development of autoimmunity through autoantigen-based immunotherapy. In this narrative review, the present possibilities for the prevention of T1D are discussed. Presently, interventions to prevent T1D are generally made in subjects in whom autoimmunity is already activated and autoantibodies against pancreatic cell components have been detected. Practically, the goal is to slow down the immune process by preserving the normal structure of the pancreatic islets for as long as possible. Unfortunately, presently methods able to avoid the risk of autoimmune activation are not available. Elimination of environmental factors associated with T1D development, reverse of epigenetic modifications that favor initiation of autoimmunity in subjects exposed to environmental factors and use of autoantigen-based immunotherapy are possible approaches, although for all these measures definitive conclusions cannot be drawn. However, the road is traced and it is possible that in a not so distant future an effective prevention of the disease to all the subjects at risk can be offered.

## Introduction

Type 1 diabetes (T1D) is a chronic autoimmune disorder that leads to progressive pancreatic ß-cell destruction and culminates in absolute insulin deficiency and stable hyperglycaemia (1). It is a relatively common disease, as the International Diabetes Federation has estimated that more than one million people <20 years of age suffer from T1D (2). It is, however, highly likely that the true incidence of T1D is significantly greater, as recent data indicate that up to 40% of new T1D cases are diagnosed in subjects older than 30 years of age (3).

Although it differs among countries, the incidence of T1D is increasing by approximately 2–3% per year, leading to several tens of thousands of new cases each year worldwide (4). Moreover, at least in some countries, a relevant increase in the number of T1D cases occurring at an early age compared to those in the past has been reported. An analysis of 29,311 new cases of T1D diagnosed in children <15 years of age in 17 European countries revealed that during the period from 1989 to 2003, the frequency of diagnosis increased by 5.4, 4.3, and 2.9% in the 0–4 year, 5–9 year, and 10–14 year age groups, respectively (5). The reasons for these epidemiological variations have not been defined. T1D is a heritable polygenic disease mainly associated with two HLA class 2 haplotypes involved in antigen presentation, although a number of HLA class I haplotypes and over 60 additional non-HLA loci associated with the risk of T1D development have been identified. However, the increase in the incidence of T1D cannot be explained by genetic drift (6). It is very likely that environmental factors play a role in triggering islet autoimmunity. This is evidenced by the increase in the number of T1D diagnoses among people who have migrated from regions with a low incidence of T1D to regions with a high incidence of T1D (7). Moreover, the incidence of T1D can significantly vary among neighboring countries where the frequency of high-risk genotypes is similar (8). Finally, although all risk genotypes have likely not yet been identified, only approximately 10–15% of individuals with genetic risk develop T1D (9).

Experimental and epidemiological studies have suggested that a number of factors including diet, vitamin D intake, infections, and gut microbiota, could play a role in favoring T1D development (10). It has been suggested that all these factors could modify gene expression through epigenetic mechanisms so inducing aberrant immune response and islet autoimmunity (11). On the other hand, epigenetic modifications have been found significantly more common in individuals with autoimmune diseases, including T1D (12). All these findings have increased our understanding of T1D pathogenesis and can be considered a potential base for the development of effective measures for T1D prevention. Interventions on the environmental factors truly associated with T1D development and/or use of measures able to restore the physiological epigenetic framework or neutralize aberrant immune response could reduce or avoid the risk of islet autoimmunity (10, 11, 13). Moreover, T1D is a continuum that progresses sequentially at variable but predictable rates through distinct identifiable stages prior to the onset of symptoms (14). Stage 1 is defined as the presence of β-cell autoimmunity as evidenced by the presence of two or more islet autoantibodies with normoglycemia and is presymptomatic, stage 2 as the presence of β-cell autoimmunity with dysglycemia and is presymptomatic, and stage 3 as onset of symptomatic disease. Theoretically, prevention of T1D, i.e., the inhibition of the autoimmune process that leads to pancreatic damage, hyperglycemia and related complications is possible. Unfortunately, which environmental factors are important as cause of autoimmunity is not precisely defined. Moreover, how to manage epigenetic mechanisms is not clearly established. Finally, methods to reduce the aberrant immune response are in the early stage of development. Although well-traced, the road to achieving an effective prevention of T1D is still long and the ultimate goal remains difficult to achieve. In this narrative review, the present knowledge in prevention of T1D is summarized.

## Environmental Factors Potentially Associated With Type 1 Diabetes (T1D) Development

### Diet in the First Months of Life

#### Gluten

Experimental studies have suggested that gluten could play a role in favoring T1D development and that the exclusion of cereals from the diet in the first phases of development could prevent the disease in a considerable portion of susceptible animals. In one of the first studies on this issue (15), a gluten-free (GF) diet was given to breeding pairs of non-obese diabetic (NOD) mice and the first generation of female NOD pups. A control group of animals with the same characteristics received a gluten-containing standard (GCS) diet. All the enrolled individuals were followed for 320 days. The incidence of T1D was significantly lower in animals on the GF diet than in mice on the GCS diet (15 vs. 64%, respectively; *p* = 0.00007). Moreover, among NOD mice receiving the GF diet that developed T1D, the disease was evidenced significantly later (244 ± 24 days) than those on the standard diet (197± 8 days; *p* = 0.03). In a more recent study (16), the offspring of NOD mice fed a GF diet during pregnancy and lactation had a reduced incidence of insulinitis and T1D compared to that in the pups of mothers receiving the GCS diet. The preventive effect was evidenced even when young animals were given the GF diet during the weaning period at 4 weeks of age. In controls at 30 weeks of age, the T1D incidence was 22% and 51% in the offspring of GF-fed mothers and those from animals given a GCS diet, respectively.

Further support for the hypothesis that gluten could favor T1D development and that a GF diet could prevent the disease was that celiac disease (CD) and T1D have several similarities. Both are autoimmune diseases, and gluten is the triggering factor for CD (17). The prevalence of CD in T1D patents is 3–8 times higher than that in the general population (18–20). Both diseases exhibit similar genetic susceptibility, as they share an association HLA DQ2 and/or DQ8 (21). NOD mice are seropositive for anti-transglutaminase antibodies (22), and when given a GF diet, they show the decreased intraepithelial infiltration of T cells, enteropathy and the incidence of autoimmune T1D compared to those in control mice (23). It has been suggested that a GF diet could decrease intestinal permeability, preventing gliadin peptides from crossing the intestinal barrier and avoiding the development of pancreatic autoimmunity. Moreover, a GF diet could dampen the innate and adaptive immune systems through the reduction of interferon (INF)-ɤ secretion from Th cells, intereukin (IL)-22 secretion from ɤδ T cell receptor-positive T cells, and the number of activated NK cells and Th17 cells. Finally, a GF diet could reduce beta-cell stress, thus preserving the number of islets (24, 25).

However, contrary to what has been derived from animal studies, the role of gluten as a trigger of T1D development in genetically susceptible children remains undefined. Because pancreatic damage in animals was prevented when gluten exposure was avoided during fetal life or in early infancy, the main aim of several human studies was to establish in which period of infant life a GF diet reduced the risk of islet autoimmunity and/or T1D development. Unfortunately, the results were conflicting and do not clarify whether a GF diet can be an effective measure to prevent T1D. A number of prospective studies carried out in genetically susceptible children did not find a consistent association between the age of the infant, the type of diet and pancreatic damage (26–30). In other studies, on the contrary, the possible role of gluten in favoring autoimmunity and/or T1D development was shown, although the impact varied slightly from study to study. Chimiel et al. prospectively followed 2,291 children with a family history of T1D from birth for 28,983 patient years (median 13.1 years) (31). They reported that the exposure of genetically susceptible subjects to gluten-containing foods before the age of 3 months was associated with a 3-fold increase in the risk of islet autoimmunity or overt T1D compared to that in children exclusively breast-fed during the first 3 months of life or given gluten between 3 and 6 months of age. In the TEDDY study, in which 8,676 children with an increased genetic risk of T1D were followed (32), the risk of islet autoimmunity was strictly related to the month of gluten introduction and progressively increased with the child's age. The hazard ratios were 0.68, 1 and 1.57 for the introduction of gluten before 4 months, between 4 and 9 months and after 9 months, respectively. No effect of a GF diet was reported in the DAISY study, in which 1,916 children with an increased risk of T1D were followed from birth to a mean age of 13.5 years (33).

No associations between age at the introduction of gluten and the development of islet autoimmunity were observed. Introduction to gluten before 4 months of age, between 4 and 5.9 months of age or at 6 months of age or later had similar adjusted hazard risks (0.97, 1, and 1.06, respectively). Moreover, the amount of gluten included in the diet between 1 and 2 years of age did not influence T1D development. However, the effect of early gluten introduction was also observed in this study. The introduction of gluten to infants before 4 months of age was associated with an increased risk of the progression from islet autoimmunity to overt T1D. A global evaluation of all these studies seemed to indicate that the importance of gluten as a trigger of T1D has not been demonstrated but that if it exists, it acts only in the first 4 months of life. This has marginal relevance for T1D prevention, as gluten-containing foods are not commonly used to feed young infants. All the official guidelines recommend that infants receive breastmilk or formula alone for the first 6 months of life (34, 35).

#### Breastfeeding and Cow Milk

More than 30 years ago, an inverse correlation was reported between breastfeeding frequency and T1D in children. Moreover, it was shown that children with T1D were breast-fed for shorter periods of time than healthy subjects or had never received breast milk (36). The protective effect of breast milk and the role of exposure to complex foreign proteins such as those in cow milk or solid food in triggering T1D development were repeatedly reported in both experimental animals and humans, suggesting that proper nutrition during the first months of life could be an effective measure for the prevention of T1D (37–41). This conclusion was confirmed by several meta-analyses and systematic reviews of human studies, although the results of individual studies do not always fully agree and sometimes suggest opposite conclusions. Norris and Scott analyzed 17 case-control studies published before 1996 and reported that the use of infant formula and cow milk before 3 months of age was associated with a moderate higher risk of T1D development than that in breast-fed children (odds ratio [OR] 1.38 and 1.61, respectively) (42). A pooled analysis of individual participant data from 43 observational studies (2 cohort and 41 case-control studies) including 9,874 patients with T1D carried out in 2012 showed that exclusive breast feeding for > 2 weeks was associated with a reduction in the risk of T1D (OR 0.75) (43). However, nonexclusive breastfeeding for >2 weeks was not protective (OR 0.93), and exclusive breastfeeding for >3 months only reduced the risk of T1D (OR 0.87). Finally, Patelarou et al. reviewed 28 studies (one cohort and 27 case-control studies) and concluded that breastfeeding for a short duration and/or a lack of breastfeeding may constitute a risk factor for the development of T1D later in life (44).

Despite these findings, no definitive conclusion regarding the potential protective effect of breast milk and the negative effect of infant formula or cow milk on the risk of T1D development has been drawn. Most of the studies included in the meta-analyses or systematic reviews had significant methodological problems mainly related to the reliability of the data regarding the duration of breastfeeding and the moment of the introduction of infant formula, cow milk or solid foods. Moreover, in most of these studies, infant nutrition was associated with overt T1D. As islet autoimmunity starts many years before the disease becomes clinically evident, it cannot be excluded that the relationship between infant feeding and T1D development seen in some studies reflects other factors that favor T1D and not those that play a role in causing the autoimmune process. However, prospective studies using hydrolyzed infant formula in genetically susceptible children did not definitively reveal the relationship between early infant feeding and T1D. A pilot study of the Trial to Reduce Insulin Dependent Diabetes Mellitus in the genetically at risk (TRIGR) in which a small number of children with an affected first-degree relative and a risk-associated HLA genotype were fed extensively with hydrolyzed casein formula in the first 6–8 months of life showed that this diet was effective in reducing the risk of T1D development in the first 10 years of life (45). However, later studies did not find a clear association. Children weaned with this formula had the same risk of T1D at 7 years of age as those receiving conventional cow milk formula (46). Similar results were obtained in another study in which follow-up was maintained for a median of 11.5 years (47).

### Vitamin D

In the last 30 years, it has been clearly shown that in addition to its classic actions on bone mineralization and growth, active vitamin D (VD) exerts several other actions that are essential for maintaining health. Among them is the modulation of innate and adaptive immunity. Practically, VD modulates the activation, maturation and apoptosis of antigen-presenting cells and T and B lymphocytes, thus generating a tolerogenic environment (48). This could explain why in NOD mice, the administration of active VD had a protective effect against T1D (49) and was accompanied by a reduction in effector T cells and an increase in Treg cells (50). Moreover, an association between VD and T1D has been suggested, because among the non-HLA susceptibility genetic markers of T1D were some VD related genetic markers, such as polymorphisms of the VD receptor gene, the VD binding protein (VDBP) gene, and the genes encoding the enzymes that metabolize VD to its active form (51). However, studies in humans have not definitively clarified when VD deficiency triggers islet autoimmunity and whether VD development and supplementation can prevent or limit islet autoimmunity.

Studies carried out to evaluate the impact of VD deficiency during fetal life have reported conflicting results. Sorensen et al. reported that lower gestational levels of active VD were associated with an increased risk of T1D development in children within the first 15 years after birth (52). Similar results were reported by Jacobsen et al., at least in male children (53). In contrast, Dong et al. did not find any statistical association between the maternal intake of VD or cod liver oil during pregnancy and the development of T1D in children (54). Finally, the level of VD in newborn infants at birth was quite similar to that in children who later developed T1D and healthy subjects (55).

Although a large number of studies have shown that patients with islet autoimmunity or T1D have significantly lower VD levels than healthy controls, low VD blood concentrations measured before the detection of islet autoantibodies were not associated with the progression to overt T1D (56). Moreover, when VD levels were prospectively measured from the 3rd month of life in a group of initially healthy children, patterns in the variation in VD concentrations were similar between those who later developed T1D and those who remained healthy, and VD values were not correlated with the age at seroconversion to autoantibody positivity (*p* = 0.79) or disease onset (*p* = 0.13) (57). However, VD supplementation during infancy seems to exert a protective effect against T1D development. Two meta-analyses concluded that the administration of VD in the first year of life may decrease the risk of T1D in later life by at least 1.5-fold (58, 59). In some studies, the effect was strictly dose-dependent, as the reduction in risk was greatest in children receiving the highest VD doses. Moreover, the timing of VD administration was critical for prevention, as the risk was reduced mainly in children who received VD in the second semester of life rather than those who received VD in the first 6 months of life (60). However, because most of the studies considered in these meta-analyses were observational, and information regarding VD intake was retrospectively collected by means of questionnaires, these conclusions clearly cannot be considered definitive, and further studies are needed to verify whether VD can truly prevent T1D development.

### Gut Microbiota

Several studies have shown that the gut microbiota composition significantly differs in NOD animals (61, 62) and humans (63) with islet autoimmunity or overt T1D compared to that in healthy subjects. Generally, the gut microbiota of T1D subjects is less diverse and less stable. In a case-control study (58), it was shown that compared to healthy subjects, patients with T1D had lower gut concentrations of *Actinobacteria* and *Firmicutes*, an increased number of *Bacteroidetes* and a reduced *Firmicutes* to *Bacteroidetes* ratio. In particular, T1D was associated with an increase in the number of *Clostridium, Bacteroides* and *Veillonella* and a significant decrease in the number of *Lactobacillus, Bifidobacterium*, the *Blautia coccoides/Eubacterium rectale* group and *Prevotella*. Moreover, the decreased abundance of *Faecalibacterium prausnitzii* (i.e., a butyrate-producing bacterium) was observed.

Interestingly, the increased diversity in the gut microbiota composition emerged in parallel with the activation of autoimmunity and the detection of autoantibodies (56). Dysbiosis can occur due to the delivery procedure and is more common in children born by cesarean section. Moreover, the diet itself, through the role of gluten and cow milk in conditioning the gut microbiota composition, can cause dysbiosis. Finally, an altered microbiota composition can derive from antibiotic treatment, which is very common in the first months of life. Dysbiosis is associated with abnormal immune system development and islet autoimmunity. The gut microbiota plays several fundamental roles in human health, including the regulation of intestinal permeability and the modulation of immune system development and activity. Animal studies have shown that germ-free mice have poorly developed intestinal and systemic lymphoid tissues and abnormal lymphocytic activity (64–66). Moreover, the gut microbiota regulates the balance between the effector branch of the immune system (CD4+ T cells) and the regulatory branch (Treg cells). Bacteria such as *Firmicutes, Lactobacilli*, and *Bifidobacteria*, which are reduced in T1D patients, are protective as they stimulate the regulatory branch of the immune system, and their presence is associated with an increased number of Treg cells. In contrast, the *Proteobacteria*, a phylum that includes *Escherichia, Salmonella, Vibrio, Helicobacter, Yersinia, Legionellales* and other pathogens frequently found in increased concentrations in the gut microbiota of T1D subjects, stimulates the effective branch of the immune system. This action is initially positive as it leads to Th1 and Th17 responses in the gut and favors the elimination of invading bacteria but can become very negative when it chronically persists (67–69).

Reverting dysbiosis to the normal gut composition can theoretically reduce the risk of T1D development. A recent study by Mariño et al. showed that feeding NOD mice a diet containing high amounts of acetate and butyrate significantly inhibited T1D onset (70). This is not surprising as some of the bacteria reduced in individuals with T1D, such as *Firmicutes and Faecalibacterium prausnitzii*, are strictly associated with the presence of butyrate and acetate, respectively (71). In addition, several animal studies have shown that probiotic administration can have promising effects on the control of T1D. An example in this was reported in a study by Dolpady et al. (72). These authors administered a mixture of several *Bifidobacteria* and *Lactobacilli* at the time of weaning and afterwards to NOD mice and found that these probiotics prevented insulitis and autoimmunity through the reduction in the number of Th1 and Th17 cells in the intestinal mucosa and pancreatic lymph nodes. Based on these studies, it has been suggested that the administration of probiotics could be a measure of T1D primary prevention.

Unfortunately, studies in humans are few and report conflicting results. In the TEDDY study, in genetically susceptible children, the administration of probiotics in the first 4 weeks of life was associated with a reduced risk of T1D development (73). In a more recent study, in contrast, the administration of a probiotic mixture given from birth to 6 months of age had exhibited no preventive effect on T1D development after 13 years or on islet cell autoimmunity after 5 years (74).

### Infections

Although there have been some exceptions (75–80), most studies that evaluated the relationship between viral infections and T1D development clearly indicated that viruses have the potential to induce islet autoimmunity and ß-cell damage and reduce insulin production, leading to full-blown T1D (81). Both DNA viruses from the *Herpesviridae* and *Parvoviridae* families and RNA viruses from the *Togaviridae, Paramyxoviridae, Retroviridae*, and *Picornaviridae* families have been associated with T1D (82). Several studies support this association. T1D is diagnosed more frequently during cold months, when several viruses circulate, than during warm months, when viral infections are less common (83, 84). Early, severe, probably viral lower respiratory infections are more frequently detected in the histories of children with T1D than in those of normal subjects (85). Viruses cause T1D in experimental animals. The D variant of encephalomyocarditis virus in mice and the Kilham rat virus in rats are paradigmatic in this regard (86).

However, among all the viruses, the most convincing data showing a strict relationship with T1D development are available for enteroviruses (EVs). Genetic studies have shown that immune responses to EVs are controlled by alleles associated with the risk of T1D. Polymorphisms in genes expressed at the β-cell and/or immune system level can lead to abnormal responses to environmental factors, such as viruses. For example, MDA5 may contribute to the pathogenesis of T1D by modifying β-cell responses to EV infection. MDA5 is a pattern recognition receptor encoded by the *IFIH1* gene that is critical for type I interferon response to EVs. In the presence of *IFIH1* gene polymorphisms, the interferon response to the viral infection of pancreatic islets is altered, and HLA class I molecules are upregulated (87), which this triggers autoimmunity (88). Several other T1D-associated genes, such as *TYK2* and *PTPN2*, exhibit an altered response to EV infection, causing a persistent, exaggerated inflammatory response and the definitive destruction of ß-cells (89). Moreover, epidemiological studies have shown that the frequency of EV infections in the general population of different European countries was inversely correlated with the incidence of T1D (90). The frequency of EV antibodies in the serum of pregnant women was higher in countries with a low or intermediate incidence of T1D than in countries with a high incidence of T1D (91). A previous EV infection was extremely common in the histories of children with recent onset T1D; EV infection was 10 times more common in children with recent onset T1D than in non-diabetic children (92). The presence of EVs in the blood of children in the first stages of T1D was more common than that in normal subjects when only islet autoimmunity was demonstrated. Finally, EV infection precedes autoimmunity activation by more than a year, thus explaining why EVs are detected in the feces of children before they develop pancreatic damage.

However, not all EVs have the same pathological potential. Coxsackieviruses (CVs) are the EVs most frequently associated with T1D, and CV serotypes A2, A4, A16, B1, and B4 are the most dangerous (93). Based on these data, it was suggested that vaccines against CV serotypes could be effective in the prevention of T1D. Theoretically, these vaccines could be useful not only in the primary prevention of T1D by avoiding infections capable of triggering pancreatic damage but also in the secondary and tertiary prevention of T1D. When administered to subjects positive for autoantibodies or with established T1D, vaccines against CV reduced the progression or worsening of the disease, respectively (94). Recombinant subunit vaccines, DNA vaccines, live attenuated vaccines, and virus-like particle vaccines against CVB3 or CVB4 have been prepared and found to induce high neutralizing antibody responses in animal models (95–99). The best-studied CV vaccine is a formalin-inactivated whole-virus vaccine prepared against CVB1. This preparation, when administered to both NOD mice and SOCS1-tg mice, induced an immune response strong enough to protect the animals from both CVB1 infection and T1D development when they were exposed to CVB1. Unvaccinated controls, on the contrary, became viraemic on day 3 post-infection and developed damage to the exocrine pancreas and lost insulin-positive ß-cells (100). However, the evidence that different EVs are associated with the development of T1D and the immune cross-reactivity between different serotypes are poor, which has led to the conclusion that a vaccine based on a single EV is inadequate to protect children from EV infection and the related risk of T1D development. To overcome this problem, at least in part, a polyvalent inactivated vaccine encompassing several CVB serotypes (PRV-101) has been studied and is presently in development (94). If effective and safe, it could represent a significant advance in the current possibilities of T1D prevention. However, adequate clinical studies are needed not only to confirm its preventive efficacy but also to exclude problems with safety and tolerability.

Theoretically, a CVB vaccine could cause T1D due to molecular mimicry, i.e., if antigens common to both ß-cells and the virus in the vaccine exist. The potential role of molecular mimicry in causing pancreatic damage has been suggested because components of EVs can cross-react with the ß-cell antigen glutamic acid decarboxylase (101), and the VP-1 protein of EVs cross-reacts with the ß-cell antigen tyrosine phosphatase IA-2/IAR (102). PRV-101 does not contain the epitope related to the ß-cell antigen glutamic acid decarboxylase. This seems to indicate that the risk of molecular mimicry with this vaccine is unlikely. Moreover, it does not include adjuvants that favor, at least in some cases, autoimmunity after vaccine administration (103).

### Other Environmental Factors

Several drugs, such as pentamidine and antibiotics, have been associated with abnormal glucose metabolism. In some cases, a direct action on pancreatic cells has been demonstrated. In others, such as in the case of streptozotocin, a N-nitroso compound, both a direct toxic mechanism and an immunologically-mediated action have been proposed (104, 105). For antibiotics, it is thought that those associated with an increased risk of T1D act through a significant modification of gut microbiota composition, so favoring emergence of bacteria associated with abnormal immune responses. Interestingly, in animal studies it was evidenced that vancomycin, that is mainly effective on Gram-positive bacteria, including those considered protective, accelerates T1D development, whereas neomycin, that mainly eliminates Gram-negative pathogens, is not associated with metabolic abnormalities (106). Pollutants, such as ozone and particulate matters <10 μm in diameter, seem associated with T1D development (107). Finally, psychological stress, through β-cell stress or direct influence on the immune system, may decrease insulin sensitivity and increase insulin resistance, so contributing to the induction or progression of diabetes-related autoimmunity (108).

## Epigenetics and Type 1 Diabetes (T1D) Development

### Epigenetic Modification in Type 1 Diabetes (T1D)

How environmental factors can favor T1D development is not precisely clarified. However, the association between some environmental factors, the emergence of autoimmune diseases and the evidence that epigenetic changes, defined as stable and heritable changes in gene expression that do not involve alterations in DNA sequence, are common among affected individuals has led to the conclusion that epigenetic alterations are a fundamental cause of autoimmune disease development, including T1D. Several mechanisms with which environmental factors can activate autoimmunity have been suggested. It is thought that chemical components of external factors can react with body components of human host, so generating new antigens against which the immune system develops specific antibodies. Moreover, it has been supposed that environmental antigens have chemical structure quite similar to that of some body constituents and for molecular mimicry autoimmunity can develop (109). The most important epigenetic modifications are DNA methylation, histone modifications and microRNA (miRNA) regulation (110). All these categories of epigenetic modifications have been found associated with insulin secretion and T1D risk, although the one most frequently detected is DNA methylation. A series of examples can clearly illustrate the potential association between some of these epigenetic modifications and emergence of conditions leading to T1D.

Hypermethylation is generally associated with gene silencing whereas hypomethylation seems to induce higher gene expression. Several studies have reported a different methylation status of genes strongly associated with T1D or its complications in patients with this disease (6, 11, 12). Consistent methylation differences between T1D patients and non-diabetic controls were found in 4 CpG sites (sites where cytosine and guanine appear consecutively on the same strand of nucleic acid) proximal to the transcription start site of the insulin gene, a gene that encodes preproinsulin and has the second highest odds ratio (OR) for T1D risk. Reduced methylation of the CpG-19, 135, and 34 sites, and increased methylation of the CpG-180 site were found (111). Variations in the ratios of circulating methylated and unmethylated insulin gene DNA have been associated with development of T1D and monitoring of methylation of β-cell-derived DNA in the blood can be considered a potential biomarker of β-cell death in T1D (112). Evaluation in 252 T1D patients and 286 age-matched controls of 6 CpGs located within the proximal promoter of interleukin 2 receptor alpha gene, a gene involved in T-reg cells, revealed that DNA methylation at CpGs−373 and−456 was significantly higher in patients than in controls (113). Moreover, several single nucleotide polymorphisms (SNPs) located in the neighboring 180 kb region and frequently detected in patients with T1D were found associated with DNA methylation at CpG−373. The evaluation of the epigenetic methylation maps of cord blood samples (114) evidenced marked differences in the methylation status of CpG sites within the major histocompatibility complex genes (cis-metQTLs) between carriers of the T1D risk haplotypes HLA-DRB1^*^03-DQA1^*^0501-DQB1^*^0201 (DR3-DQ2) and HLA-DRB1^*^04-DQA1^*^0301-DQB1^*^0302 (DR4-DQ8) compared to controls. These differences were associated with a lower HLA-DR protein expression in immune cells with the HLA-DR3-DQ2 haplotype. Finally, methylation status of CpG sites within the lactate dehydrogenase C gene was found associated with the development of insulin autoantibodies in children with the highest T1D risk genotype.

Histone modifications, i.e., modifications of proteins that allow DNA condensation into chromatin, cause alterations in chromatin stability and are followed by abnormal DNA repair, DNA replication and cell proliferation. The level of acetylation of the lysine 9 of the H3 histone protein (H3K9Ac) in the major T1D susceptibility genes HLA-DRB1 and HLA-DQB1 has been found significantly increased in patients with T1D compared to controls (115). Compared to controls, lymphocytes from patients with T1D have a significant increase in methylation level in H3K9me2 in CLTA4, a T1D susceptibility gene, involved in the regulation of T cell responses (116).

MiRNAs regulate gene expression by affecting both the stability and translation of mRNAs through direct mRNA degradation or inhibition. Epigenetic modifications of miRNA have been associated with significant modification of cell cycle and apoptosis and alterations of the immune response. Studies of miRNA expression in the Treg cells of T1D patients have shown that niRNA-146a is significantly overexpressed and miRNA 20b, 31, 99a, 100, 125b, 151, 335, and 365 are underexpressed, suggesting a direct miRNA involvement in regulation of the immune processes causing T1D (117). Moreover, several studies have reported that miRNAs are associated with β-cell dysfunction. In animals, it has been found that increased levels of miRNA-21, 34a, 146a, and 29 family can contribute to damage of pancreatic β-cells induced by proinflammatory cytokines. Overexpression of miRNAs causes impairment in glucose-induced insulin secretion and reduces expression of the transcription factor Onecut2 (118, 119). This leads to a relevant rise of granuphilin, an inhibitor of β-cell exocytosis. These findings were confirmed in humans in a study in which serum levels of miRNAs from new onset T1D children and age-matched healthy controls were compared and the miRNA expression levels were associated to β-cell function and glycemic control. Twelve upregulated human miRNAs in T1D patients (miR-152, 30a-5p, 81a, 24, 148a, 210, 27a, 29a, 26a, 27b, 25, 200a) were evidenced; several of these miRNAs were linked to apoptosis and β-cell activity. Furthermore, miRNA-25 was negatively associated with residual β-cell function (*p* = 0.0037), and a positively associated with glycemic control (HbA1c) (*p* = 0.0035) (120).

However, the best evidences that epigenetics can condition T1D development can be derived from the studies carried out in twins. There is evidence that concordance rates for the disease in monozygotic tweens is only about 50% and that discordant twins have significant differences in methylation status. The DNA methylation analysis of purified CD14+ monocytes from 15 T1D–discordant monozygotic twin pairs has evidenced hypermethylation in 54 genes and hypomethylation in 74 genes, some of which (TNF, TRAF6, CD6 and GAD2, HLADQB1, NFKB1A, respectively) are associated with T1D development (121). Similar findings were evidenced in a study in which DNA methylation profiles in B-cells DNA from monozygotic twin pairs concordant and discordant for the disease were studied. In this study significant differences in DNA methylation between children with and without disease were found in T1D associated genes HLA, INS, IL-2RB, CD226 (122). Finally, modest methylation differences between discordant monozygotic twins were recently found in other genes which are strictly associated in T1D development such as MHC, BACH2, INS-IGF2, and CLEC16A genes (123).

### Intervention for Prevention of Type 1 Diabetes (T1D)

Elimination of environmental factors associated with T1D seems the first step to obtain prevention of T1D. However, as previously highlighted, which factors play a relevant role and how, when and in which subjects they must be eliminated is not precisely defined. This does not mean that a certain degree of T1D primary prevention cannot be obtained. Following carefully the recommendation of the experts for nutrition in the first months of life, keeping in the normal range the levels of vitamin D, reducing the risk of gut dysbiosis and using, as soon as available, all the vaccines useful for preventing infections some effects can be obtained. On the other hand, following these rules can be generically useful for the preservation of health, regardless of the prevention of diabetes. Moreover, at least theoretically, prevention can be also obtained modifying epigenetic modifications associated with t1D development or inhibiting immune activity that leads to the disease. Attempts to both these goals have been made although results, even if encouraging are far, to be applied in clinical practice.

### Use of Epidrugs

Evidence that epigenetic modifications are potentially associated with T1D has led to the development of the so called epidrugs, i.e., drugs able to reverse epigenetic changes. Studies carried out in experimental animals are promising. Regarding reverse of DNA modifications, a DNA demethylating agent has been identified in 5-Aza-2′deoxycytydine (DAC). Use of low dose of this substance in mice was found able to induce demethylation of the Foxp3 gene and increase its expression in CD4(+)CD25(−) Foxp3(−) cells. Foxp3 is a key transcription factor for the development and function of Treg cells and these have a relevant immunosuppressive role. This explains why the use of DAC was effective in improving the clinical course of diabetes in cyclophosphamide-potentiated NOD mice (124).

Histone deacetylation through the use of metformin, resveratrol and fenofibrate is also considered a potential measure to control epigenetic modifications. All these drugs act as agonists of SIRT1, a nicotinamide adenine dinucleotide (NAD+)-dependent deacetylase and their use is associated with an improved glucose metabolism and increase insulin secretion in experimental animals (125–127). Effects in humans for primary T1D prevention are unknown. Addition of metformin to insulin therapy in children with T1D was associated with a reduction in insulin daily dose, body mass index and BMI z-score, although HbA1c remained substantially unmodified (128).

Positive effects were also obtained with histone acetylase inhibitors. Trichostatin A and valproic acid have been found able to improve proliferation and function of pancreatic β-cells in experimental animals with juvenile diabetes (129, 130). Finally, C66, a curcumin analog, was found able to attenuate diabetes-related increases in histone acetylation, histone acetyl transferases' activity, and the p300/CBP HAT expression, so preventing diabetic nephropathy in mice (131).

### Autoantigen-Based Immunotherapy

Autoantigen-based immunotherapy has been suggested as an effective strategy to neutralize aberrant immune responses that lead to autoimmune manifestations. In allergen-specific immunotherapy, the administration of autoantigens evoked specific immune mechanisms able to induce protective immune tolerance or anergy in already present autoreactive T cells (132). The prevention of T1D, reduced risk of the evolution of autoimmunity to overt T1D or preservation of remaining ß-cell function in cases of already manifested clinical T1D could be achieved, depending on when this type of intervention was initiated. Insulin was considered the most suitable autoantigen, as studies in NOD mice (13, 133, 134) and infants (135) have shown that autoantibodies against insulin are the first sign of islet autoimmunity and precede those against glutamic acid decarboxylase (GAD) and other pancreatic antigens. Most studies that evaluated the impact of autoantigen-based immunotherapy enrolled children after the development of autoimmunity. Their results were generally disappointing as only a minority of patients experienced a delay in T1D onset of some years (136–138).

Some data on the prevention of T1D have been collected with the Pre-POINT study (139) carried out in Germany, in which 25 islet autoantibody-negative children aged 2 to 7 years with a family history of T1D and susceptible HLA class II genotypes were enrolled. Escalating doses of oral insulin up to 67.5 mg daily were given to 15 children, whereas 10 children received a placebo for 3 to 18 months. Serum IgG levels, saliva IgA binding to insulin, and CD4+ T cell proliferative responses to insulin were periodically measured to evaluate the immune response to autoantigen administration for 18 months. Most of the children who received the highest dose of oral insulin showed a significant elevation in all the studied parameters, suggesting the protective effect of the treatment. No significant adverse events were evidenced. In particular, none of the children who received the study drug or placebo experienced hypoglycaemic episodes after the administration of medication, and no allergic reactions were observed. However, definitive conclusions could not be drawn as the study had several limitations. The number of included children was low, and only subjects with the highest genetic risk of T1D development were enrolled. The patients were significantly older than the age at the peak of islet autoantibody seroconversion. To overcome these limitations and evaluate whether insulin can be an effective measure for the prevention of T1D, a new Pre-POINT study was planned. It enrolled a large number of younger (6–24 months) genetically susceptible subjects than the previous study. The daily administration of oral insulin starting with a dose of 7.5 mg (3 months) moving to a dose of 22.5 mg (3 months) and the highest dose of 67.5 mg (6 months) was planned with the concurrent evaluation of immune response and islet autoantibody development. Presently, no results from the phase 2 clinical trial (NCT02547519) have been reported.

## Conclusions

Presently, interventions to prevent T1D are generally made in subjects in whom autoimmunity is already activated and autoantibodies against pancreatic cell components have been detected. Practically, the goal is to slow down the immune process by preserving the normal structure of the pancreatic islets for as long as possible. The prevention of T1D is significantly more important because it would completely avoid autoimmunity and preserve pancreatic integrity indefinitely.

Unfortunately, presently methods able to avoid the risk of autoimmune activation are not available. However, increase in knowledge of T1D physiopathology has identified three measures that can have a positive impact in this regard. Elimination of environmental factors associated with T1D development, reverse of epigenetic modifications that favor initiation of autoimmunity in subjects exposed to environmental factors and use of autoantigen-based immunotherapy. For all these measures, definitive conclusions cannot be drawn. However, the road is traced and it is possible that in a not so distant future an effective prevention of symptomatic disease, promotion of precision medicine, and interventions in the early stages of T1D can be offered.

## Author Contributions

SE proposed the project and revised the first draft of the manuscript. GTo wrote the first draft of the paper. GTa and ES performed the literature analysis. MB and NP critically revised the text and gave a substantial scientific contribution. All the authors approved the final version of the manuscript.

### Conflict of Interest Statement

The authors declare that the research was conducted in the absence of any commercial or financial relationships that could be construed as a potential conflict of interest.
